# Severe Cardiac and Metabolic Pathology Induced by Steroid Abuse in a Young Individual

**DOI:** 10.3390/diagnostics11081313

**Published:** 2021-07-21

**Authors:** Adrian Tirla, Cosmin Mihai Vesa, Simona Cavalu

**Affiliations:** Faculty of Medicine and Pharmacy, University of Oradea, 1 December Sq, 10, 410087 Oradea, Romania

**Keywords:** anabolic, steroids, testosterone, infarction, diabetes, dyslipidemia, ketoacidosis

## Abstract

Androgenic-Anabolic Steroids (AAS) abuse is known to play an important role in causing the systemic inflammatory response and multiple-organ dysfunction in healthy individuals. Although many of the undesirable effects of steroid abuse have been reported, at present, little is known about the effect of anabolic supplements and the correlation between cardiac and metabolic pathology. This paper presents a case of a 25 year old patient with a complex medical history after 6 months of steroid administration. Myocardial infraction, dyslipidemia, obesity, hyperuricemia, secondary diabetes, and chronic renal disease were identified after clinical and para-clinical examinations. The particularities of this case were interpreted in the context of a literature review, highlighting the effect of multi-organ damage as a result of the uncontrolled use of anabolic steroid supplements.

## 1. Introduction

Although it is restricted by law, substance abuse among adolescents represents an important public health concern. Substance use and dependence are among the most prevalent causes of adolescent morbidity and mortality in the United States. The most used substances are ethanol, nicotine, and cannabis, and 1.5% of adolescents use Androgenic-Anabolic Steroids (AAS) [1]. In the general population, a meta-analysis published in 2014 reported that 6.4% of males and 1.6% of females appealed to AAS use in their life although AAS abuse is associated with an approximately 4.6-fold higher mortality rate compared to the general population [2,3]. In a world governed by aesthetic appearance and social networks, methods for improving body composition by lowering the fat/lean mass ratio are issues of extreme interest. Regular exercising and eating a healthy and balanced diet are unfortunately not as fast rewarding as society demands, and therefore, in order to impress, some adolescents often choose methods that are not only illegal but can also put their health and lives in danger.

The general belief is that elite athletes are the biggest AAS consumers, but antidoping regulations are very strict and very few risk their careers. Surveys have shown that up to 80% of anabolic steroids use is by nonathletes, including bodybuilders and young adults [4]. AAS are synthetic derivatives of the male hormone testosterone. In normal doses and over a short time, they can improve muscle strength and increase lean body mass, but sometimes, these steroids are used in doses much higher than the recommended levels [5].

The aim of this case report is to raise awareness of the dangerous possible side effects of steroid misuse and abuse. The article exemplifies the cardiovascular, renal, and metabolic consequences of anabolic steroid administration in a healthy physically active male, in the context of a literature review. Multi-organ damage as a result of uncontrolled use of anabolic steroid supplements will be highlighted in this paper.

## 2. Case Presentation

A 25-year-old patient was brought to the emergency department for confusion, episodes of passing out, fruity-smelling breath, acute dehydration, very high blood glucose level (648 mg/dL), and an arterial blood pH of 6.9 (Table 1). A diagnosis of diabetic ketoacidosis was established, and the patient was admitted to the Diabetes Mellitus—Internal Medicine ward of Clinical County Hospital Oradea, Romania, where proper treatment for diabetic ketoacidosis was initiated.

Physical examination revealed an obnubilated, normal weight (BMI 22.3 kg/m^2^) male, with dehydrated skin, fruity-smelling breath, and polypnea. The examination of cardiovascular system revealed tachycardia combined with low blood pressure and a weak pulse.

**Figure 1:**
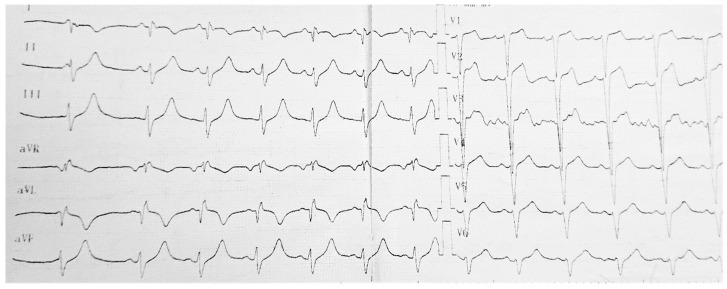
The EKG of the patient, recorded in 2015 at age 20, after consumption of AAS IV.

The patient’s past medical history was complex and revealed that at age 19, the patient, who was an amateur judo player with no recorded illness but with a family history of diabetes and cardiovascular disease (mother-hypertension, father-myocardial infraction, hypertension, and insulin-dependent diabetes mellitus type 2), was convinced by friends to take AAS in order to increase his muscular mass and sport performance. He followed the friend’s advice and took immense doses of AAS IV, alternating the following products each day: 2500 mg testosterone isocaproate, 400 mg testosterone enanthate, 500 mg Stanozolol, 1000 mg Trenbolone, and 500 mg Nadrolone. With these excessive doses, he obtained impressive effects in a short period of time. In less than 6 months, his weight increased from 80 kg to 157 kg, but a few days before finishing the 6 months cycle, he felt severe chest pain shortly after injecting the AAS. He was transported to the Emergency Room, where the EKG showed extensive anterior ST-Elevation Myocardial Infarction (STEMI), and blood samples indicated elevated cardiac necrosis biomarkers (High sensitivity cardiac troponin I (hs-cTnI)). Coronarography was proposed. He refused the procedure but remained admitted in the Cardiology department. At release, the EKG showed QS waves in the range of V1–V4 and Q waves in DI, aVL, presented in Figure 1. At the same time, the echocardiography showed severe systolic disfunction of the LV with apical LV hypokinesia and dilatation, a condition that also persisted in follow-up examinations, as shown in Table 2. Cholesterol levels were high, and dyslipidemia was also diagnosed. Chronic treatment was prescribed for the cardiovascular disease.

Three years later, at age 23, based on increased blood glucose values and specific symptoms, he was diagnosed with diabetes mellitus. The specialist recommended treatment with a basal-bolus regimen with 30–30–30 IU of rapid acting insulin before meals and 30 IU basal insulin. Unfortunately, the patient was not compliant with the treatment due to glycemic drops after rapid insulin administration and remained without any treatment for diabetes mellitus 2 years until when his condition worsened, as presented above. The fact that the patient was not compliant between 2015 and 2017 regarding his diabetes mellitus treatment is both due to the lack of interest regarding his condition but also due to an improperly prescribed insulin regimen in 2017 with very high doses of rapid-acting insulin, 30–30–30 IU/day, compared to the basal dose of 30 IU/day. In the current presentation, in December 2020, the patient was prescribed a modified insulin therapy regimen with low doses of rapid-acting insulin at a dose of 10–8–8 IU/day and a higher dose of long-acting insulin at a dose of 0–0–34 IU/day in order to avoid hypoglycemia and treatment withdrawal. The patient was also referred to a clinician psychologist in order to better understand his condition, the implications of his actions, and the importance of taking the prescribed treatment.

## 3. Paraclinical Examination

Interpretation of the case at its current evaluation is secondary diabetes complicated with diabetic ketoacidosis (DKA). Given the fact that the patient’s previous Hba1c levels were not available, the pathophysiology of his diabetes mellitus is unclear, however, given the fact that the patient has a positive family history of diabetes mellitus, our opinion is that his diabetes was accelerated by steroid consumption as well as by the patient having a very high genetic burden of cardiometabolic pathology due to his mother’s hypertension, and his father’s myocardial infraction, hypertension, and insulin-dependent diabetes mellitus type 2. Further investigations are required to exclude other differential diagnostics such as diabetes type 1 (younger age at diagnosis, ketoacidosis episodes) or type 2 diabetes (positive familial history, the presence of dyslipidemia, and hyperuricemia are relevant for metabolic syndrome).

Overall, the complete diagnostic after less than 6 months of steroid administration, over a period of 5 years was as follows: past myocardial infraction, dyslipidemia, obesity, past hyperuricemia, secondary diabetes, and chronic renal disease. All of these features are, in fact, the most feared known effects that may occur after AAS administration.

## 4. Discussion

Adverse effects of AAS are known and well documented. Since antiquity, different testicular extracts have been used in order to promote virilization, although effects were mostly placebo due to the low hormonal concentration of the product and the inactivation of orally taken testosterone in first liver pass [6]. A breakthrough was made in 1935, when testosterone was synthetized for the first time, and since then, it has been used along with other steroids to treat gonadal disfunctions [7]. Illicit use was also a fact, and side effects began to appear. Table 3 includes some of the most noticed adverse effects of steroid abuse.

As shown above, steroid supplementation is not harmless or without unwanted effects. The cardiovascular system is affected by the promotion of atherogenesis, hypercoagulability, and increased myocardial oxygen requirements caused by hypertrophy. Kaşikçioğlu et al. studied the effect of steroids on the cardiac system, presenting cases of myocardial infarction [9]. Chang et al. reviewed the implication of AAS in coagulation, thrombus formation, and fibrinolysis, demonstrating that almost all coagulation factor concentrations are modified after steroid administration [11]. Several studies and case reports presented AAS induced direct myocardial injury, and the most common pathological finding in autopsied hearts revealed LV hypertrophy, frequently associated with fibrosis and myocytolysis [14,15,16]. Even if complications may be more frequent in AAS users suffering acute myocardial infraction (AMI), AAS-related cardiac events are expected to be underreported in the medical literature considering the socio-psychological aspects and the intention to hide AAS use, both for legal reasons and social stigmatization.

In this context, Table 4 presents 20 examples of acute myocardial infarction after AAS abuse. The multiple key components of the cardiovascular system are affected by these substances. Several studies have demonstrated alteration in lipid metabolism after AAS administration, consisting of rising LDL cholesterol and lowering HDL cholesterol concentration, leading to dyslipidemia, one of the most important elements in aterogenesis and cardiovascular disease [3,57,58,59,60,61]. Glazer et al. reviewed the effect of lowering HDL cholesterol and the increase of insulin resistance caused by AAS, concluding that AAS consumption can lead to an increase in cardiovascular risk that is more than 6 times higher compared to the general population [8].

Regarding blood pressure and endothelial function, other important risk factors in cardiovascular disease, the effects of AAS are controversial. Although some animal model studies have demonstrated the capacity of AAS to lower blood pressure by increasing NO synthetase activity [62,63], multiple clinical studies have demonstrated an increase in both systolic and diastolic blood pressure values after AAS administration [14,64,65].

This explanation may lay in the fact that at high doses, the effect of nitric oxide is neutralized by Reactive Oxygen Species (ROS) generated by increased oxidative stress, resulting in vasospasms combined with high sodium retention [65,66].

Hypercoagulability is a reality after AAS administration and can be explained by the increase in hemoglobin concentration, thromboxane A2, and fibrinogen synthesis, while prostacyclin production is inhibited [11,12,13,21,67,68,69,70].

**Table 4:** Examples of acute myocardial infarction after AAS abuse.

Case Nr	Patient	Type of Steroid Consumption	Negative Effect	Comorbidities/Associated Treatment	Ref.
1.	39-year-old man	testosterone enanthate 500 mg intramuscularly every 2 weeks	Acute Myocardial Infarction-LAD artery	HIV zidovudine 300 mgtwice/day, lamivudine 150 mg twice/day, and indinavir 800 mg every 8 h, all orally. albuterol inhaler	[71]
2.	25-year-old male	Nandrolone decanoate 100–200 mg	Acute Myocardial Infarction-proximal LAD artery	none	[72]
3.	61-year-old	metenolone enanthate (45 mg)	Acute Myocardial Infarction-RCA	Diabetes, hypertension Aplastic anemia	[73]
4.	24-year-old bodybuilder	Stanozol 40 mg orally Nadrolone 200 mg intramuscularly twice a week Sustanon 250 intramuscularly weekly	Acute Myocardial Infarction Dyslipidemia	none	[74]
5.	59-year-old female	metenolone enanthate (100 mg) oxymetholone (30 mg)	Acute Myocardial Infarction-RCA	Secondary glucose intolerance Aplastic anemia	[73]
6.	31-year-old man	Multiple AAS cycles	Acute Myocardial Infarction-distal RCA	Crohn’s disease-infliximab	[75]
7.	41-year-old male	oxymetholone and methenolone	acute inferior myocardial infarction RCA proximal large renal infarction	none	[76]
8.	27-year-old	Not specified	Acute Myocardial Infarction-LAD	none	[77]
9.	41-year-old male	Not specified/more than 20 years of use	acute inferior myocardial infarction RCA arrhythmias with variable atrioventricular blocks acute kidney injuryacute liver injury	none	[78]
10.	24-year-old male	stanozolol, testosterone, tamoxifen, mesterolone, and nandrolone	Death Thrombosis LCA LAD Cardiomegaly	precordial pain	[79]
11.	26-year-old physically active male	Sustanon 250 mg, once per week for 6 months	acute inferior myocardial infarction LAD ostium occlusion	none	[80]
12.	31-year-old	Several AAS including enanthate, decanoate, and sipanate	Acute Myocardial Infarction-totally occluded RCA	none	[81]
13.	25-year-old Caucasian male	oxandrolone, 40 mg/day (daily); clenbuterol, 0.08 mg/day (daily); mesterolone, 50 mg/day (daily); hGH, 10 IU/day (daily); nandrolone, 600 mg/day (twice a week); testosterone cypionate, 400 mg/day (twice a week); stanozolol, 100 mg/day (thrice a week); drostanolone, 200 mg/day (thrice a week); trenbolone at 200 mg/day (thrice a week); testosterone propionate, 100 mg/day (thrice a week); boldenone, 400 mg/day (twice a week); and methenolone, 200 mg/day (twice a week)	Posteroinferior Acute Myocardial Infarction-RCA stenosis	none	[82]
14.	26-year-old male	trenbolone acetate, stanozolol, and testosterone.	Acute Myocardial Infarction-LAD	Peptic gastric disease 8 years before	[83]
15.	26-year-old male	Stanozolol 2 mL each week, Inj Testosterone 1 mL each week, and oral T3 (triiodothyronine) 25 mcg each day	Acute Myocardial Infarction-90% proximal LAD occlusion	hepatitis A 2 years before	[84]
16.	25-year-old man	testosterone	Acute Myocardial Infarction-proximal LAD right renal artery thrombosis/embolus	none	[85]
17.	38-year-old African American mal	Not specified	Acute Myocardial Infarction-proximal LAD occlusion	none	[86]
18.	30-year-old male	oral testosterone for several years	Acute Myocardial Infarction-LAD stenosis	none	[87]
19.	23-year-old body builder male	Trenbolone Acetate	Acute Myocardial Infarction-LAD and LCX stenosis	none	[88]
20.	50-year-old body-builder Caucasian man	nandrolone and erythropoietin	Acute Myocardial Infarction-LAD thrombosis	none	[89]

Vascular disease and hypercoagulability lead to microcirculatory disfunction in sensible organs such as the heart, brain, and kidney. Parente et al. unraveled the pathophysiology behind the kidney injury due to AAS use [22]. miR-21 and miR-205 are newly identified and useful biomarkers that can be used to detect the potential damage of AAS consumption on kidney tissue, including fibrotic changes connected to their known adverse effects on renal and cardiovascular function [90,91].

A total of nine case reports exemplified in Table 5 presented renal injury in conjunction with cardiac disorders after AAS use. Although there is strong evidence on the relationship between AAS and kidney injury, the pathophysiological mechanisms behind it are multiple and intricate. AAS are thought to have a direct nephrotoxic effect that, when combined with hyperfiltration, cause high creatine levels, leading to focal segmental glomerulosclerosis [92]. On other side, secondary to cholestasis caused by AAS, bile acid nephropathy has been shown to cause acute kidney injury (AKI) [93]. Last, but not to be forgotten, are hypercoagulability and polycythemia, which are secondary to AAS administration and have been proven to cause renal infarction/thrombosis in multiple cases [76,85]. The high protein diet followed by body builders should also be taken into consideration. Most of the time, AAS supplementations is associated with high levels of protein isolates and concentrates. There is enough evidence in the literature to prove the harmful effects on the kidney’s glomerular filtration rate (GFR) caused by high protein intake [94,95,96].

The particularity of the case presented in our paper is the development of secondary diabetes. Testosterone is known to increase insulin sensitivity to lower the glycemic index, while testosterone deficit can lead to metabolic syndrome and diabetes [102,103,104]. However, only a few pieces of evidence suggest AAS to be the cause of diabetes development. Table 6 presents briefly two cases of diabetes presenting after consumption of AAS and growth hormone. The causative effect of AAS alone is not powerful in these two cases, as growth hormone is known to raise the blood sugar levels. In a study with 100 participants, Rasmusssen et al. demonstrated lowering insulin sensitivity among AAS users [105], while Geraci et al. suggests that androgens significantly affect insulin sensitivity [55]. Further investigations are required to determine the exact dose-metabolic effect of AAS, as most of the reported studies are limited to the recommended dose, and many consumers exceed these values. Even if there is little evidence directly linking AAS and diabetes, these substances influence some risk of the factors for diabetic disease, such as hypertension, increased body weight, dyslipidemia, and dysfunctions in other systems that can alter the metabolic balance.

This aim of our paper was to raise awareness of the real danger represented by the ease of access to different AAS formulations. While elite athletes are subjected to rigorous antidoping testing in conformity with World Anti-Doping Agency (WADA) regulations, the general population, especially young people, is just a few clicks away from receiving an entire pharmacy of AAS [106,107]. Besides the original packaged products, a black market of “home bottled” products exists, where the final product combination, doses, and sterilization are vaguely known.

However, technology advances the role of miRNA are gaining importance because the negative effects of AAS can be detected in different tissues, and these miRNAs can serve as biomarkers of AAS doping abuse, given the fact that AAS induces significant negative effects on gene expression and therefore on cellular function as well [108,109].

Adolescents represent an easy target for this type of products in their desire to show off and impress. The case presented above is a clear example of what happens if AAS are administered without medical consultation. Living with myocardial infraction and diabetes beginning in the early 20s is a serious chronic health condition. The economic implications are hard to estimate, but they should also be taken in consideration. The burden on the health system from this type of patient is heavy and long lasting. A national and international strategy should be considered in order to limit the general population’s accessibility to this type of substance.

## 5. Conclusions

The use of AAS represents a serious public health issue. As exemplified above, steroids can and will cause immediate or long-term side effects, especially considering that most consumers exceed the recommended doses. It are young healthy people who are at risk. The abuse of AAS drugs has been linked to many pathological conditions, such as acute myocardial infarction, dyslipidemia, hypertension, hepatic dysfunction, kidney injury, infertility, metabolic, neurologic, and psychiatric disorders. We suggest that long-term AAS abuse predisposes young people to multiple organ dysfunction syndromes. The particularity of the case presented in this paper is the development of secondary diabetes as a result of AAS consumption.

## Figures and Tables

**Table 1:** A comparison of the laboratory results recorded at the time of hospitalization.

Test	October 2015	April 2017	December 2020	UM	Normal Values
White blood cells (WBC)	20.13	11.60	26.65	10^3^/μL	4.0–10.0
Neutrophils (NEU)	17.51	7.87	16.28	10^3^/μL	2.4–6.5
Lymphocytes (LYM)	1.58	2.39	8.978	10^3^/μL	1.0–4.0
Monocytes (Mono)	0.76	0.43	1.001	10^3^/μL	0.3–1.0
Red blood cells (RBC)	4.68	5.53	5.106	10^6^/μL	3.8–5.1
Hematocrit (HCT)	48.49	52.77	49.92	%	35–47
Hemoglobin (HGB)	17.52	16.97	16.59	g/dL	13.2–17.3
pH	-	-	6.9		7.35–7.45
Serum creatinine	0.96	1.11	1.95	mg/dL	0.10–1.2
Glycemia	122	95	648	mg/dL	65–115
Hemoglobin A1c (HbA1C)	-	-	14.7	%	4–6
Glomerular filtration rate (GFR)	-	-	34.28	mL/min/1.73 m^2^	>90 mL/min/1.73 m^2^
Uric acid	6.9	7.8	3.6	mg/dL	3.5–7.2
Aspartate aminotransferase (AST/GOT)	52	19	12	U/L	5–34
Alanine aminotransferase (AST/GOT)	77	26	20	U/L	0–55
Bilirubin	0.65	0.75	0.45	mg/dL	0.2–1.2
Cholesterol (CHOL)	216	201	146	mg/dL	0–199
HDL CHOL	54	38	32	mg/dL	40–60
High sensitive Troponin I	103,252.5	-	-	pg/ml	–
Urine glucose	-	-	≥1000 mg/dL	mg/dL	negative
Urine proteins	-	-	50 mg/dL	mg/dL	negative
Urine ketones	-	-	100 mg/dL	mg/dL	negative

**Table 2:** Echocardiographic evaluation: acute and follow up.

Year/Anatomical Area	2015	2017	2020
Aortic anulus	25 mm	24 mm	29 mm
Ascendent aorta	32 mm	40 mm	37 mm
Left atrium	36 mm	35 mm	36 mm
Interventricular sept	13 mm Akinesia 2/3 apical	11.2 apical Akinesia	16 mm septal hypokinesia
Left ventricle	With moderate wall hypertrophy, 1/3 basal hypokinesia 2/3 apical akinesia	20 mL apical aneurisms	apical hypokinesia
left ventricular ejection fraction	30%	44%	45%
General Observation/Valvular Disfunction	1–2 mm pericardial effusion	Arrythmia Mitral regurgitation grade I Aortic regurgitation grade I	Mitral regurgitation grade II Aortic regurgitation grade I

**Table 3:** Negative effects of steroid consumption on different systems.

System	Effect	Reference
Cardiovascular	Dyslipidemia	[8]
	Myocardial infarction	[9]
	Hypertension	[10,11]
	Thrombosis/thromboembolism	[11,12,13]
	Aortic Dissection	[14]
	Myocardial hypertrophy/LVH	[15]
	Dilatative cardiopathy/heart failure	[16,17]
	Arrhythmia	[18]
	Sudden death	[19]
Hematological	Polycythemia	[20]
	Hypercoagulability	[21]
Renal	Renal failure	[22]
Hepatic	hepatic adenoma hepatocellular carcinoma	[23]
	Peliosis	[24]
	Hepatotoxicity	[25]
	Steatosis	[26]
	Cholestasis	[27]
Musculoskeletal	Short stature (Premature epiphyseal closure)	[28,29]
	Tendon ruptures	[30]
	Rhabdomyolysis	[31,32]
Neurological	Neurotoxicity	[33,34]
	Dementia	[35]
Psychiatric	Aggressiveness, Criminal behavior antisocial behavior	[36,37,38,39]
	Delirium, mania	[40]
	Suicidal behavior	[41]
Dermatological	Acne	[42]
	Alopecia	[43]
	Hirsutism	[44]
	Stretch marks (striae distensae)	[45]
Male reproductive system	Anabolic steroid induced Hypogonadism (ASIH)	[46,47]
	Infertility	[48]
	Erectile dysfunction	[49]
	Gynecomastia	[50]
Female reproductive system	Infertility	[51]
	Breast atrophy	[52]
	Voice deepening	[53]
Metabolic disorders	Relative energy deficiency in sport syndrome RED-S	[51]
	Hypoleptinemia	[54]
	DiabetesInsulin resistance	[55,56]

**Table 5:** Examples of kidney injury after AAS abuse.

Case Nr	Patient	Type of Steroid Consumption	Negative Effect	Comorbidities/Associated Treatment	Ref.
1.	41-year-old male	oxymetholone and methenolone	acute inferior myocardial infarction RCA proximal large renal infarction	none	[76]
2.	41-year-old male	Not specified/more than 20 years of use	acute inferior myocardial infarction RCA arrhythmias with variable atrioventricular blocks acute kidney injury acute liver injury	none	[78]
3.	25-year-old man	testosterone	Acute Myocardial Infarction-proximal LAD right renal artery thrombosis/embolus	none	[85]
4.	30-yr-old white male pr	testosterone, methyl-1-testosterone [taken orally], growth hormone, and insulin	Nephrotic syndromeFocal Segmental Glomerulosclerosis	none	[93]
5.	43-year-old male	trenbolone acetate testosterone	left renal parenchymal infarct and acute kidney injury	OCD escitalopram 20 mg	[97]
6.	28 years	Methandienone 10–50 mg Stanozolol 50 mg	Acute kidney injury Acute liver injury	none	[98]
7.	33-year-old man	Oxymetholone methadone, tramadol, opium	Acute kidney injury	drug dependence borderline personality disorder	[99]
8.	31-year-old man	Chloromethylandrostenediol 50 mg Epitiostanol 54 mg	Acute kidney injury Acute liver injury	none	[100]
9.	26-year-old male	Stanozolol	Cholestasis Acute renal failure	none	[101]

**Table 6:** Cases of diabetes presenting after consumption of AAS and growth hormone.

Case Nr	Patient	Type of Steroid Consumption	Negative Effect	Comorbidities/Associated Treatment	Ref.
1.	33-year-old male	bovine growth hormone and testosterone	diabetes	none	[55]
2.	36-year-old male	Multiple including growth hormone, Testosterone propionate Testosterone enanthate Stanozolol Trenbelone acetate	Diabetes	none	[105]

## Data Availability

Not applicable.

## References

[1] Johnston L., O’Malley P., Bachman J., Schulenberg J. (2010). Monitoring the Future: National Survey Results on Drug Use, 1975–2009. Volume II: College Students and Adults Ages 19–50.

[2] Sagoe D., Molde H., Andreassen C.S., Torsheim T., Pallesen S. (2014). The global epidemiology of anabolic-androgenic steroid use: A meta-analysis and meta-regression analysis. Ann. Epidemiol..

[3] Kanayama G., Hudson J.I., Pope H.G. (2008). Long-term psychiatric and medical consequences of anabolic-androgenic steroid abuse: A looming public health concern?. Drug Alcohol Depend..

[4] Parkinson A.B., Evans N.A. (2006). Anabolic androgenic steroids: A survey of 500 users. Med. Sci. Sports Exerc..

[5] Trenton A.J., Currier G.W. (2005). Behavioural manifestations of anabolic steroid use. CNS Drugs.

[6] Nieschlag E., Cüppers H.J., Wickings E.J. (1977). Influence of sex, testicular development and liver function on the bioavailability of oral testosterone. Eur. J. Clin. Investig..

[7] Nieschlag E., Nieschlag S., Hohl A. (2017). The history of testosterone and the testes: From antiquity to modern times. Testosterone.

[8] Glazer G. (1991). Atherogenic effects of anabolic steroids on serum lipid levels: A literature review. Arch. Intern. Med..

[9] Kaşikçioğlu E. (2005). Anabolic-androgenic steroids: A bad tenor for cardiovascular orchestra (Myocardial infarction with intracoronary thrombus induced by anabolic steroids). Anadolu Kardiyol. Derg..

[10] Maravelias C., Dona A., Stefanidou M., Spiliopoulou C. (2005). Adverse effects of anabolic steroids in athletes. Toxicol. Lett..

[11] Chang S., Münster A.-M., Gram J., Sidelmann J. (2018). Anabolic androgenic steroid abuse: The effects on thrombosis risk, coagulation, and fibrinolysis. Semin. Thromb. Hemost..

[12] Lippi G., Banfi G. (2011). Doping and Thrombosis in Sports. Semin. Thromb. Hemost..

[13] McCulloch N.A., Abbas J.R., Simms M.H. (2014). Multiple arterial thromboses associated with anabolic androgenic steroids. Clin. J. Sport. Med..

[14] Heydari A., Asadmobini A., Sabzi F. (2020). Anabolic Steroid Use and Aortic Dissection in Athletes: A Case Series. Oman Med. J..

[15] Far H.R.M., Ågren G., Thiblin I. (2012). Cardiac hypertrophy in deceased users of anabolic androgenic steroids: An investigation of autopsy findings. Cardiovasc. Pathol..

[16] Montisci M., El Mazloum R., Cecchetto G., Terranova C., Ferrara S.D., Thiene G., Basso C. (2012). Anabolic androgenic steroids abuse and cardiac death in athletes: Morphological and toxicological findings in four fatal cases. Forensic Sci. Int..

[17] Kasikcioglu E., Oflaz H., Umman B., Bugra Z. (2009). Androgenic anabolic steroids also impair right ventricular function. Int. J. Cardiol..

[18] Medei E., Marocolo M., de Carvalho Rodrigues D., Arantes P.C., Takiya C.M., Silva J., Rondinelli E., Coeli dos Santos Goldenberg R., de Carvalho A.C.C., Nascimento J.H.M. (2010). Chronic treatment with anabolic steroids induces ventricular repolarization disturbances: Cellular, ionic and molecular mechanism. J. Mol. Cell. Cardiol..

[19] Lehmann S., Thomas A., Schiwy-Bochat K.-H., Geyer H., Thevis M., Glenewinkel F., Rothschild M.A., Andresen-Streichert H., Juebner M. (2019). Death after misuse of anabolic substances (clenbuterol, stanozolol and metandienone). Forensic Sci. Int..

[20] Stergiopoulos K., Mathews R., Brennan J., Setaro J., Kort S. (2008). Anabolic steroids, acute myocardial infarction and polycythemia: A case report and review of the literature. Vasc. Health Risk Manag..

[21] Kahn N.N., Sinha A.K., Spungen A.M., Bauman W.A. (2006). Effects of Oxandrolone, an Anabolic Steroid, on Hemostasis. Am. J. Hematol..

[22] Parente Filho S.L.A., de Carvalho Gomes P.E.A., Forte G.A., Lima L.L.L., da Silva Júnior G.B., Meneses G.C., Martins A.M.C., Daher E.D.F. (2020). Kidney disease associated with androgenic–anabolic steroids and vitamin supplements abuse: Be aware!. Nefrología.

[23] Hui C.L., Lee Z.J. (2019). Hepatic disorders associated with exogenous sex steroids: MR imaging findings. Abdom. Radiol..

[24] Neri M., Bello S., Bonsignore A., Cantatore S., Riezzo I., Turillazzi E., Fineschi V. (2011). Anabolic Androgenic Steroids Abuse and Liver Toxicity. Mini Rev. Med. Chem..

[25] Bond P., Llewellyn W., Van Mol P. (2016). Anabolic androgenic steroid-induced hepatotoxicity. Med. Hypotheses.

[26] Schwingel P.A., Cotrim H.P., Salles B.R., Almeida C.E., dos Santos C.R., Nachef B., Andrade A.R., Zoppi C.C. (2011). Anabolic-androgenic steroids: A possible new risk factor of toxicant-associated fatty liver disease: Anabolic steroids and TAFLD. Liver Int..

[27] (2012). LiverTox: Clinical and Research Information on Drug-Induced Liver Injury.

[28] Matsuo K., Fujieda K. (2006). Excessive androgens and short stature in childhood. Clin. Calcium.

[29] Richman R.A., Kirsch L.R. (1988). Testosterone treatment in adolescent boys with constitutional delay in growth and development. N. Engl. J. Med..

[30] Marqueti R.C., Paulino M.G., Fernandes M.N., de Oliveira E.M., Selistre-de-Araujo H.S. (2014). Tendon structural adaptations to load exercise are inhibited by anabolic androgenic steroids: AAS administration changes tendon structure. Scand. J. Med. Sci. Sports.

[31] Benjamin A., Anderson A., Zrelec S. (2020). Delayed rhabdomyolysis secondary to anabolic-androgenic steroid use. Clin. Med..

[32] Farkash U., Shabshin N., Pritsch M. (2009). Rhabdomyolysis of the deltoid muscle in a bodybuilder using anabolic-androgenic steroids: A case report. J. Athl. Train..

[33] Pomara C., Neri M., Bello S., Fiore C., Riezzo I., Turillazzi E. (2015). Neurotoxicity by synthetic androgen steroids: Oxidative stress, apoptosis, and neuropathology: A review. Curr. Neopharmacol..

[34] Bertozzi G., Salerno M., Pomara C., Sessa F. (2019). Neuropsychiatric and behavioral involvement in AAS abusers. A literature review. Medicina.

[35] Kaufman M.J., Kanayama G., Hudson J.I., Pope H.G. (2019). Supraphysiologic-dose anabolic–androgenic steroid use: A risk factor for dementia?. Neurosci. Biobehav. Rev..

[36] Christoffersen T., Andersen J.T., Dalhoff K.P., Horwitz H. (2019). Anabolic-androgenic steroids and the risk of imprisonment. Drug Alcohol Depend..

[37] Vaskinn A., Hauger L.E., Bjørnebekk A. (2020). Theory of mind in users of anabolic androgenic steroids. Psychopharmacology.

[38] Coccaro E.F. (2017). Testosterone and aggression: More than just biology?. Biol. Psychiatry.

[39] Carré J.M., Geniole S.N., Ortiz T.L., Bird B.M., Videto A., Bonin P.L. (2017). Exogenous testosterone rapidly increases aggressive behavior in dominant and impulsive men. Biol. Psychiatry.

[40] Khoodoruth M.A.S., Khan A.A. (2020). Anabolic steroids-induced delirium: A case report. Medicine.

[41] Gestsdottir S., Kristjansdottir H., Sigurdsson H., Sigfusdottir I.D. (2020). Prevalence, mental health and substance use of anabolic steroid users: A population-based study on young individuals. Scand. J. Public Health.

[42] Hassoun L., Chahal D., Sivamani R., Larsen L. (2016). The use of hormonal agents in the treatment of acne. Sem. Cutan. Med. Surg..

[43] Piraccini B.M., Alessandrini A. (2014). Androgenetic alopecia. G. Ital. Dermatol. Venereol..

[44] Lizneva D., Gavrilova-Jordan L., Walker W., Azziz R. (2016). Androgen excess: Investigations and management. Best Pract. Res. Clin. Obstet. Gynaecol..

[45] Wollina U., Pabst F., Schönlebe J., Abdel-Naser M.B., Konrad H., Gruner M., Haroske G., Klemm E., Schreiber G. (2007). Side-effects of topical androgenic and anabolic substances and steroids. A short review. Acta Dermatovenerol. Alp. Pannonica Adriat..

[46] Christou M.A., Christou P.A., Markozannes G., Tsatsoulis A., Mastorakos G., Tigas S. (2017). Effects of anabolic androgenic steroids on the reproductive system of athletes and recreational users: A systematic review and meta-analysis. Sports Med..

[47] Boregowda K., Joels L., Stephens J.W., Price D.E. (2011). Persistent primary hypogonadism associated with anabolic steroid abuse. Fertil. Steril..

[48] Ohlander S.J., Lindgren M.C., Lipshultz L.I. (2016). Testosterone and male infertility. Urol. Clin. N. Am..

[49] Horwitz H., Andersen J.T., Dalhoff K.P. (2019). Health consequences of androgenic anabolic steroid use. J. Intern. Med..

[50] Basaria S. (2010). Androgen abuse in athletes: Detection and consequences. J. Clin. Endocrinol. Metab..

[51] Nieschlag E., Vorona E. (2015). Mechanisms in endocrinology: Medical consequences of doping with anabolic androgenic steroids: Effects on reproductive functions. Eur. J. Endocrinol..

[52] Goyal A., Malhotra R., Kulshrestha V., Kachhawa G. (2019). Severe hyperandrogenism due to ovarian hyperthecosis in a young woman. BMJ Case Rep..

[53] Zeng L.T., Han B., Liu B.L., Chen X., Zhu H., Chen Y., Chen M., Liu J.H., Liu Y., Qiao J. (2019). Clinical features and genetic characteristics of 33 patients with simple virilizing form of 21-hydroxylase deficiency. Zhonghua Nei Ke Za Zhi.

[54] Hislop M., Ratanjee B., Soule S., Marais A. (1999). Effects of anabolic-androgenic steroid use or gonadal testosterone suppression on serum leptin concentration in men. Eur. J. Endocrinol..

[55] Geraci M.J., Cole M., Davis P. (2011). New onset diabetes associated with bovine growth hormone and testosterone abuse in a young body builder. Hum. Exp. Toxicol..

[56] Allan C. (2014). Sex steroids and glucose metabolism. Asian J. Androl..

[57] García-Esperón C., Hervás-García J.V., Jiménez-González M., Pérez de la Ossa-Herrero N., Gomis-Cortina M., Dorado-Bouix L., López-Cancio Martinez E., Castaño-Duque C.H., Millán-Torné M., Dávalos A. (2013). Ingestion of anabolic steroids and ischaemic stroke. A clinical case report and review of the literature. Rev. Neurol..

[58] Kopin L., Lowenstein C.J. (2017). Dyslipidemia. Ann. Intern. Med..

[59] Hartgens F., Rietjens G., Keizer H.A., Kuipers H., Wolffenbuttel B.H. (2004). Effects of androgenic-anabolic steroids on apolipoproteins and lipoprotein (a). Br. J. Sports Med..

[60] Kindermann W. (2006). Cardiovascular side effects of anabolic-androgenic steroids. Herz.

[61] Baldo-Enzi G., Giada F., Zuliani G., Baroni L., Vitale E., Enzi G., Magnanini P., Fellin R. (1990). Lipid and apoprotein modifications in body builders during and after self-administration of anabolic steroids. Metabolism.

[62] Perusquía M., Greenway C.D., Perkins L.M., Stallone J.N. (2015). Systemic hypotensive effects of testosterone are androgen structure-specific and neuronal nitric oxide synthase-dependent. Am. J. Physiol. Reg. Integr. Compar. Physiol..

[63] Janjic M.M., Stojkov N.J., Andric S.A., Kostic T.S. (2012). Anabolic–androgenic steroids induce apoptosis and NOS2 (nitric-oxide synthase 2) in adult rat Leydig cells following in vivo exposure. Reprod. Toxicol..

[64] Solberg E.E., Borjesson M., Sharma S., Papadakis M., Wilhelm M., Drezner J.A., Harmon K.G., Alonso J.M., Heidbuchel H., Dugmore D. (2016). Sudden cardiac arrest in sports—Need for uniform registration: A position paper from the Sport Cardiology Section of the European Association for Cardiovascular Prevention and Rehabilitation. Eur. J. Prev. Cardiol..

[65] Ammar E.M., Said S.A., Hassan M.S. (2004). Enhanced vasoconstriction and reduced vasorelaxation induced by testosterone and nandrolone in hypercholesterolemic rabbits. Pharmacol. Res..

[66] Arazi H., Mohammadjafari H., Asadi A. (2017). Use of anabolic androgenic steroids produces greater oxidative stress responses to resistance exercise in strength-trained men. Toxicol. Rep..

[67] Alén M. (1985). Androgenic steroid effects on liver and red cells. Br. J. Sports Med..

[68] Houghton D.E., Alsawas M., Barrioneuvo P., Tello M., Farah W., Beuschel B., Prokop L.J., Layton J.B., Murad M.H., Moll S. (2018). Testosterone therapy and venous thromboembolism: A systematic review and meta-analysis. Thromb. Res..

[69] Ferenchick G.S. (1991). Anabolic/androgenic steroid abuse and thrombosis: Is there a connection?. Med. Hypotheses.

[70] Choe H., Elfil M., De Sancho M.T. (2016). Inherited antithrombin deficiency and anabolic steroids: A risky combination. Blood Coagul. Fibrinolysis.

[71] Varriale P., Mirzai-Tehrane M., Sedighi A. (1999). Acute myocardial infarction associated with anabolic steroids in a young HIV-infected patient. Pharmacotherapy.

[72] Huie M.J. (1994). An acute myocardial infarction occurring in an anabolic steroid user. Med. Sci. Sports Exerc..

[73] Toyama M., Watanabe S., Kobayashi T., Iida K., Koseki S., Yamaguchi I., Sugishita Y. (1994). Two cases of acute myocardial infarction associated with ap1astic anemia during treatment with anabolic steroids. Jpn. Heart J..

[74] Kennedy C. (1993). Myocardial infarction in association with misuse of anabolic steroids. Ulster Med. J..

[75] Zhang Q., Shan K.S., Raza A., Manda N., Nace T. (2020). A rare case report and literature review of anabolic-androgenic steroids (AAS)-induced acute myocardial infarction. Cureus.

[76] Ilhan E., Demirci D., Güvenç T.S., Calık A.N. (2010). Acute myocardial infarction and renal infarction in a bodybuilder using anabolic steroids. Turk. Kardiyol. Dern. Ars..

[77] Peoples K., Kobe D., Campana C., Simon E. (2014). Hyperhomocysteinemia-induced myocardial infarction in a young male using anabolic steroids. Am. J. Emerg. Med..

[78] Flo F.J., Kanu O., Teleb M., Chen Y., Siddiqui T. (2018). Anabolic androgenic steroid–induced acute myocardial infarction with multiorgan failure. Baylor Univ. Med. Center Proc..

[79] Hernández-Guerra A.I., Tapia J., Menéndez-Quintanal L.M., Lucena J.S. (2019). Sudden cardiac death in anabolic androgenic steroids abuse: Case report and literature review. Forensic Sci. Res..

[80] Alrabadi N., Jarrah M., Alzoubi K. (2020). Acute myocardial infarction with cardiogenic shock in a young physically active physician concurrently using the anabolic steroid sustanon: A case report. Biomed Rep..

[81] Wysoczanski M., Rachko M., Bergmann S.R. (2008). Acute myocardial infarction in a young man using anabolic steroids. Angiology.

[82] Santos R.P., Pereira A., Guedes H., Lourenço C., Azevedo J., Pinto P. (2015). Anabolic drugs and myocardial infarction—A clinical case report. Arq. Bras. Cardiol..

[83] Melhem A.J., Araújo A.C., Figueiredo F.N.S., Figueiredo D.L.A. (2020). Acute myocardial infarction in a young bodybuilder: A case report and review of the literature. Am. J. Case Rep..

[84] Jain V., Goel G. (2020). Acute myocardial infarction in young newbie bodybuilder using multiple steroid and protein supplements. J. Cardiol. Cases.

[85] Tan B.E.X., Chowdhury M., Hall C., Baibhav B. (2020). Exogenous testosterone abuse and myocardial infarction in a young bodybuilder. Am. J. Med..

[86] Samreen F., Popal U., Qutrio Baloch Z.A. (2021). Anabolic steroid-induced myocardial infarction in a young male. Cureus.

[87] Major R.W., Pierides M., Squire I.B., Roberts E. (2015). Bodybuilding, exogenous testosterone use and myocardial infarction. QJM.

[88] Shahsavari Nia K., Rahmani F., Ebrahimi Bakhtavar H., Hashemi Aghdam Y., Balafar M. (2014). A young man with myocardial infarction due to trenbolone acetate: A case report. Emergency.

[89] Lunghetti S., Zacà V., Maffei S., Carrera A., Gaddi R., Diciolla F., Maccherini M., Chiavarelli M., Mondillo S., Favilli R. (2009). Cardiogenic shock complicating myocardial infarction in a doped athlete. Acute Card. Care.

[90] Sessa F., Salerno M., Bertozzi G., Cipolloni L., Messina G., Aromatario M., Polo L., Turillazzi E., Pomara C. (2020). miRNAs as novel biomarkers of chronic kidney in-jury in anabolic-androgenic steroid users: An experimental study. Front. Pharmacol..

[91] Moisi M.I., Rus M., Bungau S., Zaha D.C., Uivarosan D., Fratila O., Tit D.M., Endres L., Nistor-Cseppento D.C., Popescu M.I. (2020). Acute coronary syndromes in chronic kidney disease: Clinical and therapeutic characteristics. Medicina.

[92] Herlitz L.C., Markowitz G.S., Farris A.B., Schwimmer J.A., Stokes M.B., Kunis C., Colvin R.B., D’Agati V.D. (2010). Development of focal segmental glomerulosclerosis after anabolic steroid abuse. J. Am. Soc. Nephrol..

[93] Luciano R.L., Castano E., Moeckel G., Perazella M.A. (2014). Bile acid nephropathy in a bodybuilder abusing an anabolic androgenic steroid. Am. J. Kidney Dis..

[94] Ko G.J., Obi Y., Tortorici A.R., Kalantar-Zadeh K. (2017). Dietary protein intake and chronic kidney disease. Curr. Opin. Clin. Nutr. Metab. Care.

[95] Ko G.-J., Rhee C.M., Kalantar-Zadeh K., Joshi S. (2020). The effects of high-protein diets on kidney health and longevity. J. Am. Soc. Nephrol..

[96] Jhee J.H., Kee Y.K., Park S., Kim H., Park J.T., Han S.H., Kang S.-W., Yoo T.-H. (2019). High-protein diet with renal hyperfiltration is associated with rapid decline rate of renal function: A community-based prospective cohort study. Nephrol. Dial. Transpl..

[97] Colburn S., Childers W.K., Chacon A., Swailes A., Ahmed F.M., Sahi R. (2017). The cost of seeking an edge: Recurrent renal infarction in setting of recreational use of anabolic steroids. Ann. Med. Surg..

[98] Habscheid W., Abele U., Dahm H.H. (2008). Schwere cholestase mit nierenversagen durch anabolika bei einem bodybuilder. Dtsch. Med. Wochenschr..

[99] Tarashandefoumani A., Elyasi F. (2018). Oxymetholone-induced acute renal failure: A case report. Caspian J. Intern. Med..

[100] Flores A., Nustas R., Nguyen H.-L., Rahimi R.S. (2016). Severe cholestasis and bile acid nephropathy from anabolic steroids successfully treated with plasmapheresis. ACG Case Rep. J..

[101] Yoshida E.M., Karim M.A., Shaikh J.F., Soos J.G., Erb S.R. (1994). At what price, glory? Severe cholestasis and acute renal failure in an athlete abusing stanozolol. Can. Med. Assoc. J..

[102] Kelly D.M., Jones T.H. (2013). Testosterone: A metabolic hormone in health and disease. J. Endocrinol..

[103] Saad F., Gooren L. (2009). The role of testosterone in the metabolic syndrome: A review. J. Steroid Biochem. Mol. Biol..

[104] Yassin A., Haider A., Haider K.S., Caliber M., Doros G., Saad F., Garvey W.T. (2019). Testosterone therapy in men with hypogonadism prevents progression from prediabetes to type 2 diabetes: Eight-year data from a registry study. Dia Care.

[105] Rasmussen J.J., Schou M., Selmer C., Johansen M.L., Gustafsson F., Frystyk J., Dela F., Faber J., Kistorp C. (2017). Insulin sensitivity in relation to fat distribution and plasma adipocytokines among abusers of anabolic androgenic steroids. Clin. Endocrinol..

[106] Young J., Anwar A., Milne C. (2007). Strong diabetes * Commentary. Br. J. Sports Med..

[107] Cordaro F.G., Lombardo S., Cosentino M. (2011). Selling androgenic anabolic steroids by the pound: Identification and analysis of popular websites on the Internet: Androgenic anabolic steroids and the Internet. Scand. J. Med. Sci. Sports.

[108] Sessa F., Salerno M., Cipolloni L., Bertozzi G., Messina G., Mizio G.D., Asmundo A., Pomara C. (2020). Anabolic-androgenic steroids and brain injury: miRNA evaluation in users compared to cocaine abusers and elderly people. Aging.

[109] Sessa F., Salerno M., Di Mizio G., Bertozzi G., Messina G., Tomaiuolo B., Pisanelli D., Maglietta F., Ricci P., Pomara C. (2018). Anabolic Androgenic Steroids: Searching New Molecular Biomarkers. Front. Pharmacol..

